# Nrf2/HO-1 signaling activation alleviates cigarette smoke-induced inflammation in chronic obstructive pulmonary disease by suppressing NLRP3-mediated pyroptosis

**DOI:** 10.1186/s13019-024-02530-3

**Published:** 2024-02-05

**Authors:** Yanan Zhang, Jinxia Wang, Yuling Wang, Kai Lei

**Affiliations:** 1 Department of Respiratory and Critical Care Medicine, General Hospital of Ningxia Medical University, 804 Shengli South Street, Yinchuan, 750004 China; 2 Ningxia Medical University, Yinchuan, China

**Keywords:** COPD, Inflammation, Nrf2/HO-1 pathway, NLRP3, Pyroptosis

## Abstract

**Background:**

This study examined the effect of the nuclear factor erythroid 2-related factor 2 (Nrf2)/heme oxygenase 1 (HO-1) pathway on chronic obstructive pulmonary disease (COPD) and the potential molecular mechanism.

**Methods:**

A COPD mouse model was established by cigarette smoke exposure and administered with either ML385 or dimethyl fumarate (DMF). Airway resistance of mice was detected. IL-1β and IL-6 levels in mice alveolar lavage fluid were examined by enzyme-linked immunosorbent assay. Hematoxylin and eosin staining and immunohistochemical of lung tissues were utilized to detect lung injury and NLRP3 expression. DMF was used to treat COPD cell model constructed by exposing normal human bronchial epithelial (NHBE) cells to cigarette smoke extract. NHBE cells were transfected by NLRP3-expression vectors. Expression of proteins was detected by Western blot.

**Results:**

COPD mice showed the enhanced airway resistance, the inactivated Nrf2/HO-1 pathway and the overexpressed NLRP3, Caspase-1 and GSDMD-N proteins in lung tissues, and the increased IL-1β and IL-6 levels in alveolar lavage fluid. ML385 treatment augmented these indicators and lung injury in COPD mice. However, DMF intervention attenuated these indicators and lung injury in COPD mice. Nrf2/HO-1 pathway inactivation and overexpression of NLRP3, Caspase-1 and GSDMD-N proteins were observed in COPD cells. DMF intervention activated Nrf2/HO-1 pathway and down-regulated NLRP3, Caspase-1 and GSDMD-N proteins in COPD cells. However, NLRP3 overexpression abolished the effect of DMF on COPD cells.

**Conclusion:**

Nrf2/HO-1 pathway activation may alleviate inflammation in COPD by suppressing the NLRP3-related pyroptosis. Activating the Nrf2/HO-1 pathway may be an effective method to treat COPD.

## Introduction

Chronic obstructive pulmonary disease (COPD) is characterized by consistent airway inflammation and irreversible airflow obstruction, which is the third leading cause of mortality in the world [1, 2]. COPD is mainly triggered by the inhalation of toxic particles, especially tobacco smoke and polluted air [3]. Some COPD patients are caused by genetic factors, lung infection and malformation of lung growth and development [4, 5]. In terms of pathogenesis, oxidative stress and inflammatory activation are two well-recognized factors that induce chronic airway inflammation and lung parenchyma destruction [6]. As the further understanding of COPD pathogenesis, some molecular targeted drugs have been demonstrated to exert well effects on relieving COPD, such as antioxidants, cytokine-targeted drugs and some inhibitors of enzyme and signaling pathways [4]. However, COPD is still incurable, which brings serious adverse impact on the life quality of patients and substantial economic burden for public health concern [7]. A better understanding of the molecular mechanisms underlying COPD will be conducive to the development of effective treatment strategies.

Recent studies have been found that cigarette smoke(CS) induced reactive oxygen species (ROS) and inflammation response are closely related to the pyroptosis which involved in the development of COPD [8, 9]. Pyroptosis is a type of programmed cell death mediated by the inflammatory caspases. The release of cell contents caused by pyroptosis will aggravate the inflammatory response to further deteriorate COPD [10]. Pyroptosis represents a unique cell death form usually activated by the NOD‑like receptor protein‑3 (NLRP3) inflammasome and Caspase‑1 [10]. The activated NLRP3 inflammasome can not only exacerbate the inflammatory response, but also can induce the hydrolysis and activation of Caspase‑1 to cleavage the pyroptosis effector protein gasdermin D (GSDMD). After cleavage, GSDMD N-terminal domain (GSDMD-N) protein was generated and then inserted into the surface of the cell membrane to form holes, which finally causes the expansion and rupture of cells and the ultimately inflammatory reaction [11]. Previous study has been implied that the activated NLRP3 can lead to the Caspase-1 mediated release of proinflammatory factors such as interleukin-18 (IL-18) and IL-1β to trigger the inflammatory outburst in COPD mice [12]. However, whether NLRP3 contributes the development of COPD by inducing pyroptosis has not yet been definitively elucidated.

It has been identified that the increased level of CS induced ROS in COPD can exacerbate the inflammatory responses by directly activating the NLRP3 inflammasomes [13]. It is well known that the nuclear factor erythroid 2-related factor 2 (Nrf2)/heme oxygenase 1 (HO-1) pathway is one of the most critical intracellular pathways to block the reactive oxygen species. Nrf2 can exert the antioxidant role by enhancing the expression of HO-1 protein [14]. Accumulating studies have indicated that the activated Nrf2 pathway can alleviate the inflammatory response and improve lung function in COPD [15]. Interestingly, a recent study has been revealed that the Nrf2 pathway activation can relieve rheumatoid arthritis by suppressing the pyroptosis via blocking the activation of NLRP3 [16]. Unfortunately, whether Nrf2 can alleviate pyroptosis and inflammatory response in COPD by suppressing NLRP3 remains unclear.

Therefore, this study hypothesized that the activated Nrf2/HO-1 pathway might alleviate inflammation in COPD by suppressing the NLRP3-related pyroptosis. This article may provide new molecular target for COPD treatment.

## Materials and methods

### Animals and construction of COPD model

C57BL/6 male mice (n = 48, 8 weeks old) were commercially provided by Junke Biological Engineering (Nanjing, Jiangsu, China), and maintained in a non-specific pathogen room at 22 °C with free access to food and water. The day/night cycle was 12 h. Animal experiments were implemented after being ratified by the Ethics Committee of General Hospital of Ningxia Medical University (2020–276). Mice were randomly divided into four groups: Control group (n = 12), COPD group (n = 12), COPD + ML385 group (n = 12) and COPD + DMF group (n = 12).

Mice of the Control group inhaled room air without cigarette smoke (CS) exposure. Mice of the COPD group were subjected to the construction of COPD model by exposing to CS (nine cigarettes/h, 2 h per exposure, twice per day, six days per week) in a whole-body exposure chamber for 90 days [17]. The cigarettes were purchased from Guangdong Tobacco Industry (Guangzhou, China), and each cigarette contained 1.0 mg nicotine, 11 mg tar and 13 mg carbon monoxide per cigarette. For mice of the COPD + ML385 group and the COPD + DMF group, they firstly experienced CS exposure, and then administered with either ML385 (an inhibitor of Nrf2, 30 mg/kg [18] per time, once every 2 days, for 2 weeks) or dimethyl fumarate (DMF) (an activator of Nrf2, 80 mg/kg [19] per time, once every 2 days, for 2 weeks) by gavage. In this research, the construction of COPD mouse model was done at Ningxia Medical University, and the model was constructed with the technical support and guidance of Zhonghong Boyuan Biotechnology Co., Ltd (Jiangxi, China).

### Airway resistance detection

The lung function of mice was evaluated by detecting the airway resistance according to previously reported [20]. Briefly, after being deeply anesthetized by 60 mg/kg sodium pentobarbital, mice were tracheostomized with a catheter. Subsequently, mice were paralyzed by intraperitoneal injection of rocuronium (10 mg/mL, 50 mL) to deprive them of independent respiration. Lung function test system for laboratory animals (PFT-MR, TOW Intelligent Technology Co., Ltd, Shanghai, China) was used for the airway resistance detection by nebulization to a dose of methacholine 25 mg/mL in phosphate buffer solution. The airway resistance detection was done at Zhonghong Boyuan Biotechnology Co., Ltd (Jiangxi, China).

### Enzyme-linked immunosorbent assay (ELISA)

Mice were deeply anesthetized with 60 mg/kg sodium pentobarbital and then endotracheal intubated. A total of 600 μL normal saline was injected into the bronchus through the catheter to collect the alveolar lavage fluid. The alveolar lavage fluid experienced 10 min centrifugation at 1500 rpm/min and 4 °C. The supernatant of the alveolar lavage fluid was harvested [21]. The levels of IL-1β and IL-6 in the supernatant was detected by using the IL-1β ELISA kit (JK-E4183, Jingkang Biological Engineering, Shanghai, China) and IL-6 ELISA kit (JK-E3123, Jingkang Biological Engineering, Shanghai, China). The detection process was carried out in line with the directions.

### Histological staining

After being deeply anesthetized, mice were killed through neck dislocation to harvest the whole lung tissues. Hematoxylin and eosin (H&E) staining of the lung tissues was implemented to examine the pathological changes. Briefly, the lung tissues were cut into sections (5 μm) and sequentially stained with by hematoxylin and eosin solution by using the HE staining kit (Yubo Biotechnology, Shanghai, China) strictly according to the manufacturer's instructions. After routinely dehydration, the sections were enclosed in neutral resin and observed under a light microscope (Olympus, Tykyo, Japan).

Immunohistochemical (IHC) staining was performed to research the expression of NLRP3 protein in lung tissues. Briefly, the lung sections were immersed into 3% H_2_O_2_ for 10 min and then boiled in citric acid buffer for 3 min. The sections were then blocked by normal goat serum (Lianmai Biotechnology, Shanghai, China) for 30 min at 37 °C, probed by rabbit anti-NLRP3 primary antibody (1:100, K008087P, Solarbio, Beijing, China) for 12 h at 4 °C, and then reacted with goat anti rabbit secondary antibody (1:200, SE134, Solarbio, Beijing, China) for 30 min at 37 °C. After 3, 3-diaminobenzidine (DAB, Solarbio, Beijing, China) and hematoxylin counterstaining, the sections were routinely dehydrated, sealed and then observed under a light microscope (Olympus, Tykyo, Japan).

### Preparation of cigarette smoke extract (CSE)

The preparation of CSE was performed according to previously reported [22]. The cigarette smoke with a volume of 400 mL was collected and thoroughly mixed with 20 mL non-serum RPMI 1640. The mixture was considered as 100% concentration of CSE. After being adjusted to pH 7.4, the mixture was filtered by a 0.22 μm filter. The absorbance value of the mixture was detected between 0.9 and 1.2 by UV spectrophotometer (756MC/756CRT, Shanghai Chromatographic Instrument, Shanghai, China). The mixture was diluted to 5% concentration of CSE before using.

### Cell transfection

Normal human bronchial epithelial (NHBE) cells were purchased from the American Type Culture Collection (ATCC, Manassas, VA, USA), and cultivated in RPMI1640 (Solarbio, Beijing, China) suspended with 10% fetal bovine serum (FBS, Solarbio, Beijing, China), 100 U/mL streptomycin (Solarbio, Beijing, China) and 100 U/mL penicillin (Solarbio, Beijing, China) at 37 °C and 5% CO_2_.

NHBE cells were suspended into serum-free RPMI1640, plated into 6-well plates (1 × 10^6^ cells/mL per well), and transfected by either pCDNA3.1-NLRP3 vectors (served as the NLRP3 group) or pCDNA3.1 vectors (named the NC group) via using Lipofectamine 3000 (Thermo Fisher Scientific, Shanghai, China). The transfection was implemented following the manufacturer’s instruction. pCDNA3.1-NLRP3 vectors and pCDNA3.1 vectors were all commercially provided by GeneChem (Shanghai, China).

### Establishment of COPD cell model and treatment

For the induction of the COPD cell model, NHBE cells were grown in the 6-well plates with serum-free RPMI1640 containing 5% CSE for 24 h at 37 °C and 5% CO_2_ [22] (named the CSE group).

Moreover, RPMI1640 containing 10% FBS and 20 μM [19] DMF (Hengfei Biotechnology, Shanghai, China) was utilized to pretreat NHBE cells for 2 h at 37 °C and 5% CO_2_. These cells then underwent incubation by serum-free RPMI1640 containing 5% CSE for 24 h 37 °C and 5% CO_2_. These cells were set as the CSE + DMF group.

For NHBE cells of the CSE + DMF + NLRP3 group, they were firstly transfected by pCDNA3.1-NLRP3 vectors (GeneChem, Shnghai, China) as described in the “Cell transfection” section, then cultured for 2 h in RPMI1640 containing 10% FBS and 20 μM DMF at 37 °C and 5% CO_2_, and finally incubated in serum-free RPMI1640 containing 5% CSE for 24 h at 37 °C and 5% CO_2_. NHBE cells without any treatment were used as Control group. After the relevant treatment, NHBE cells of each group were harvested for Western blot analysis.

### Western blot

Total proteins in cells and lung tissues were extracted by using RIPA buffer suspended with protease inhibitor (Yeasen Biotechnology, Shanghai, China). BCA assay kit (ZY80815, Zeye Biotechnology, Shanghai, China) was for the determination of total protein concentration. Each total protein sample (50 μg) was subjected to sodium dodecyl sulfate–polyacrylamide gel electrophoresis, and then transferred onto polyvinylidene difluoride (PVDF) membranes. The proteins were blocked by 5% skimmed milk (for 1 h at room temperature), incubated by rabbit anti-primary antibody (for 12 h at 4 °C), and then treated by secondary antibody treatment (for 2 h at room temperature). The protein blots were developed by enhanced chemiluminescence reagent (Yeasen Biotechnology, Shanghai, China), and qualified by the Image J software (version 1.43, NIH, Bethesda, MD, USA).

The primary antibodies were: anti-Nrf2 (1:1000, ab92946, Abcam, Shanghai, China), anti-HO-1 (1:1000, ab13243, Abcam, Shanghai, China), anti-NLRP3 (1:1000, ab263899, Abcam, Shanghai, China), anti-Caspase-1 (1:1000, ab74279, Abcam, Shanghai, China), anti-GSDMD-N (1:1000, ab155233, Abcam, Shanghai, China), anti-β-actin (1:1000, ab8227, Abcam, Shanghai, China). The secondary antibody was horseradish peroxidase-labeled goat anti-rabbit secondary antibody (1:2000, 111-035-003, Yanhui Biotechnology, Shanghai, China).

### Statistical analysis

All experiments were independently repeated in triplicate. The statistical analysis of data (shown as mean ± standard deviation) was implemented by GraphPad Prism 6.0 software (GraphPad Software Inc., San Diego, CA, USA). Student’s t-test was utilized for the data comparison between two groups. One-way analysis of variance (ANOVA) with Tukey’s *post-hoc* test was applied for the data comparison in more than two groups. *P* < 0.05 meant a statistically significant difference.

## Results

### The inactivated Nrf2/HO-1 signaling and the enhanced pyroptosis and inflammatory response in COPD mice

**Fig. 1:**
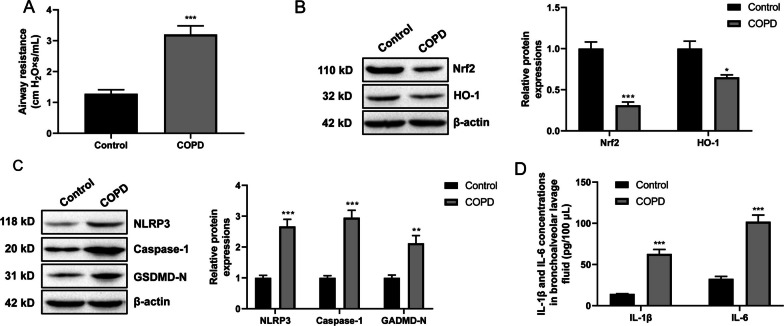
The inactivated Nrf2/HO-1 pathway and the enhanced pyroptosis and inflammatory response in COPD mice. **A** Lung function test revealed the enhanced airway resistance of COPD mice. **B**, **C** Western blot of lung tissues indicated the inactivated Nrf2/HO-1 pathway (**B**) and the enhanced expression of NLRP3 and pyroptosis-related proteins (**C**) in COPD mice. **D** ELISA of the alveolar lavage fluid implied the increased level of proinflammatory factors (IL-1β and IL-6) in COPD mice. *P < 0.05, **P < 0.01 and ***P < 0.001 vs. Control group

Lung function test showed the intensified airway resistance of COPD mice (the COPD group) than normal mice (the Control group) (*P* < 0.001) (Fig. 1A). Western blot of lung tissues exhibited the reduced expression of Nrf2 and HO-1 proteins in mice of the COPD group, when compared to the Control group (*P* < 0.05, *P* < 0.001) (Fig. 1B). Simultaneously, the expression of pyroptosis-related proteins, including NLRP3, Caspase-1 and GSDMD-N, was explored. As a result, the elevated expression of NLRP3, Caspase-1 and GSDMD-N proteins was occurred in the lung tissues of the COPD group relative to the Control group (*P* < 0.01, *P* < 0.001) (Fig. 1C). In the alveolar lavage fluid, higher levels of IL-1β and IL-6 were observed in mice of the COPD group, in comparison to the Control group (*P* < 0.001) (Fig. 1D). It suggested that the Nrf2/HO-1 pathway was inactivated and the pyroptosis and inflammatory response was augmented in COPD mice.

### The activated Nrf2/HO-1 pathway relieved the lung injury and inflammatory response in COPD mice

**Fig. 2:**
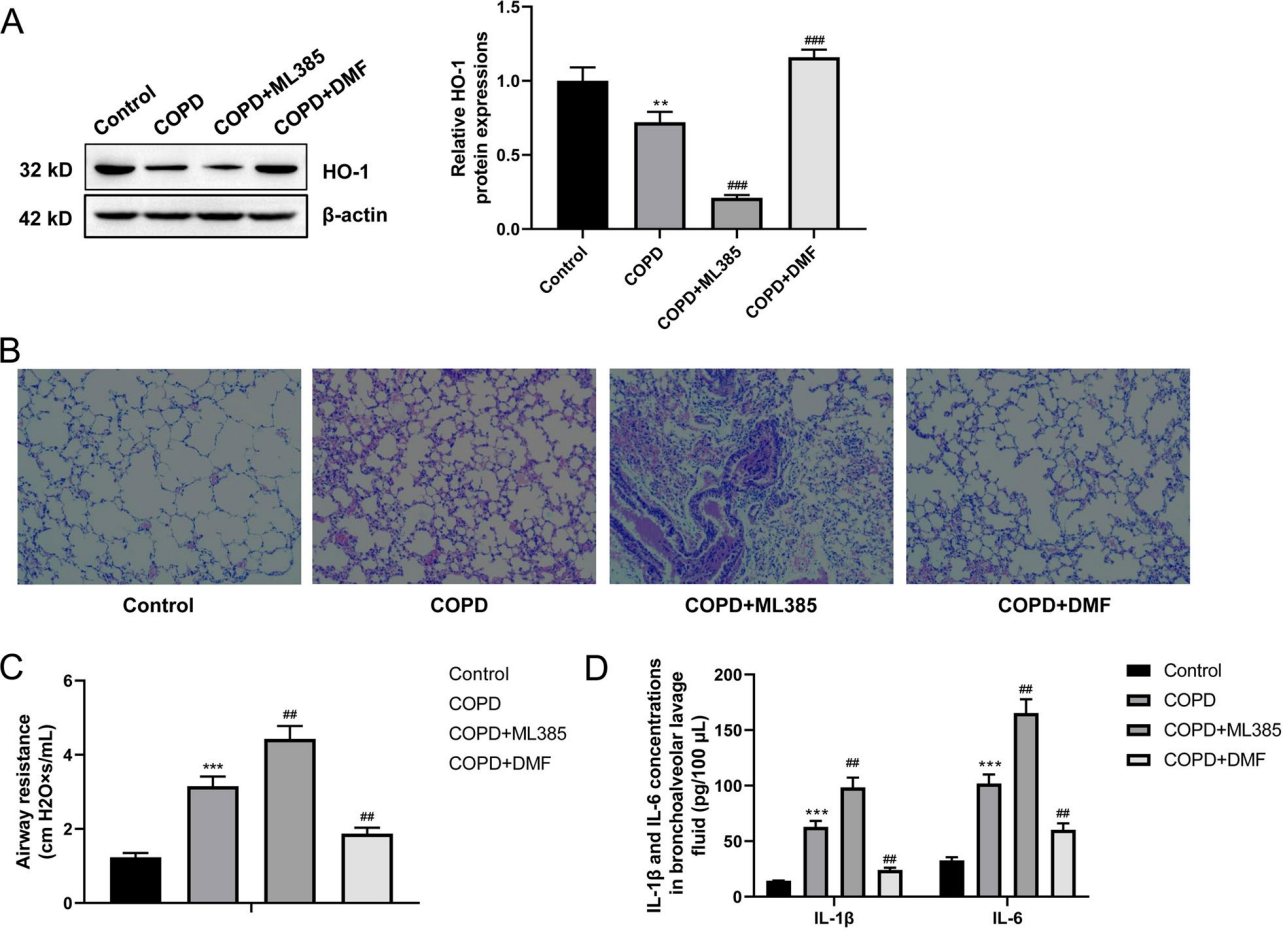
The activated Nrf2/HO-1 pathway relieved the lung injury and inflammatory response in COPD mice. **A** Western blot of lung tissues indicated the inactivated Nrf2/HO-1 pathway by ML385 treatment and activated Nrf2/HO-1 pathway by DMF intervention in COPD mice. **B** H&E staining of the lung tissues suggested the relieved lung injury and inflammatory infiltration by activating the Nrf2/HO-1 pathway in COPD mice. **C** Lung function test revealed the reduced airway resistance by activating the Nrf2/HO-1 pathway in COPD mice. **D** ELISA of the alveolar lavage fluid revealed the decreased proinflammatory factors (IL-1β and IL-6) by activating the Nrf2/HO-1 pathway in COPD mice. **P < 0.01 and ***P < 0.001 vs. Control group. ##* P* < 0.01 and ### *P* < 0.001 vs. COPD group

Next, we used either ML385 (an inhibitor of the Nrf2/HO-1 pathway) or DMF (an activator of the Nrf2/HO-1 pathway) to treat COPD mice. As presented in Fig. 2A, the lower expression of HO-1 protein was occurred in the lung tissues of COPD mice than normal mice (*P* < 0.01). However, matched to the COPD group, mice of the COPD + ML385 group expressed lower HO-1 protein in the lung tissues (*P* < 0.001), whereas mice of the COPD + DMF group expressed higher HO-1 protein (*P* < 0.001). This revealed that the Nrf2/HO-1 pathway activity was effectively regulated in lung tissues of COPD mice by ML385 or DMF treatment.

H&E staining of the lung tissues showed the intensified lung injury and inflammatory infiltration in mice of the COPD group when compared to the Control group. In comparison to the COPD group, mice lung injury and inflammatory infiltration of the COPD + ML385 group was augmented but the COPD + DMF group was relieved (Fig. 2B). Additionally, the enhanced airway resistance of mice in the COPD group was observed when relative to the Control group (*P* < 0.001). ML385 treatment significantly exacerbated the airway resistance of COPD mice (*P* < 0.01), while DMF intervention remarkably attenuated it (*P* < 0.01) (Fig. 2C). ELISA of the alveolar lavage fluid exhibited the increased levels of IL-1β and IL-6 in COPD mice than normal mice (*P* < 0.001). Interestingly, ML385 treatment intensified the levels of IL-1β and IL-6 in mice alveolar lavage fluid (*P* < 0.01), but DMF treatment showed the opposite effect (*P* < 0.01) (Fig. 2D). All of these data implied that the activation of the Nrf2/HO-1 pathway could relieve the lung injury and inflammatory response in COPD mice.

### The activated Nrf2/HO-1 pathway suppressed the pyroptosis in lung tissues of COPD mice

**Fig. 3:**
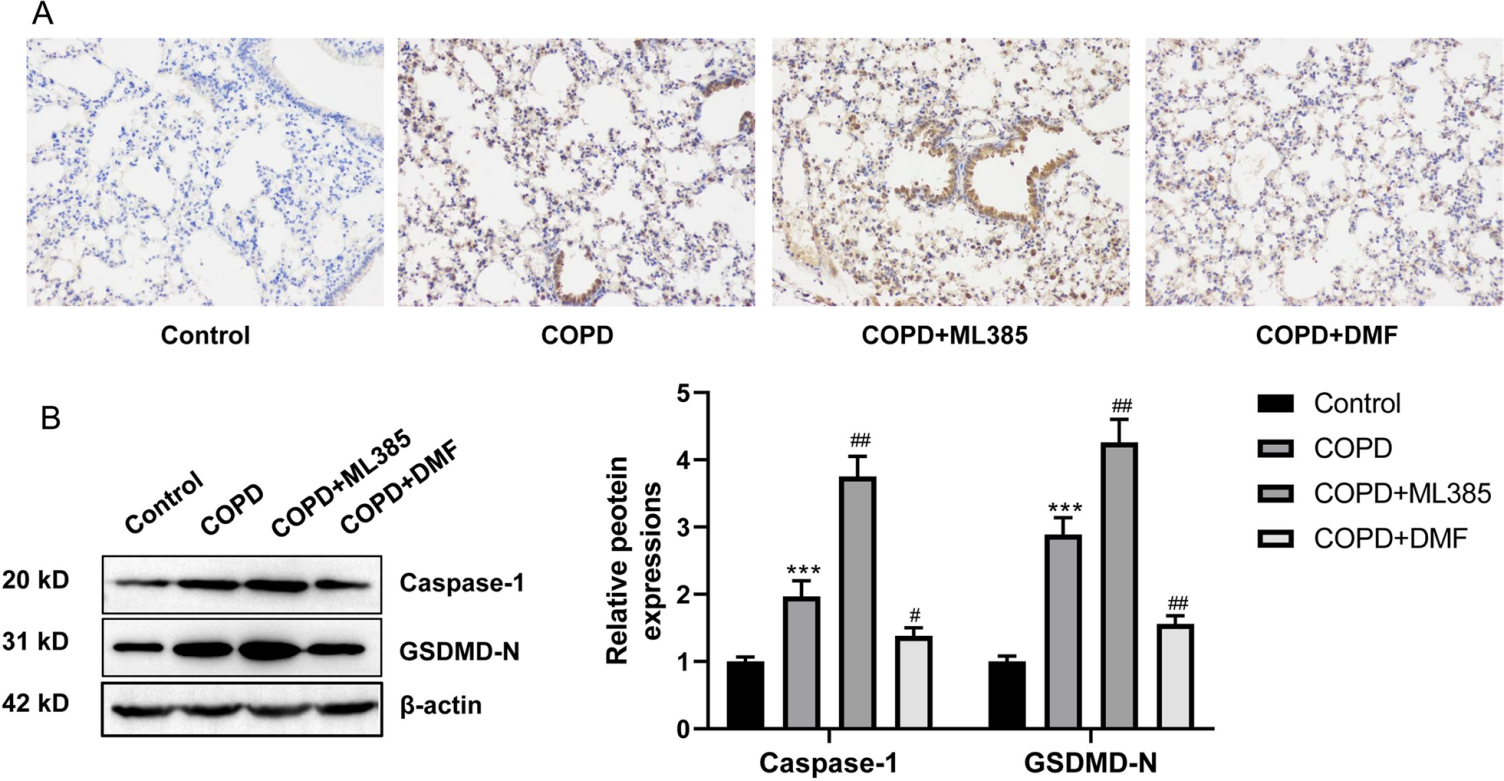
The activated Nrf2/HO-1 pathway suppressed the pyroptosis in lung tissues of COPD mice. **A** IHC staining of the lung tissues revealed that the activated Nrf2/HO-1 pathway suppressed NLRP3 expression in COPD mice. **B** Western blot illustrated that the activated Nrf2/HO-1 pathway reduced the expression of pyroptosis-related proteins (Caspase-1 and GSDMD-N) in lung tissues of COPD mice. ***P < 0.001 vs. Control group. #* P* < 0.05 and ##* P* < 0.01 vs. COPD group

IHC staining of the lung tissues showed the intensified NLRP3 expression in COPD mice than normal mice. ML385 treatment enhanced the expression of NLRP3 in lung tissues of COPD mice. However, DMF intervention exerted the opposite effect, which attenuated NLRP3 expression in lung tissues of COPD mice (Fig. 3A). Results of Western blot presented the higher expressed Caspase-1 and GSDMD-N proteins in COPD mice than normal mice (*P* < 0.001). Interestingly, ML385 treatment further increased the expression of Caspase-1 and GSDMD-N proteins in lung tissues of COPD mice (*P* < 0.01). Conversely, DMF intervention diminished the expression of Caspase-1 and GSDMD-N proteins in lung tissues of COPD mice (*P* < 0.05, *P* < 0.01) (Fig. 3B). Thus, the activated Nrf2/HO-1 pathway could suppress the pyroptosis in lung tissues of COPD mice.

### NLRP3 overexpression abolished the suppression of the activated Nrf2/HO-1 pathway on COPD cell pyroptosis

**Fig. 4:**
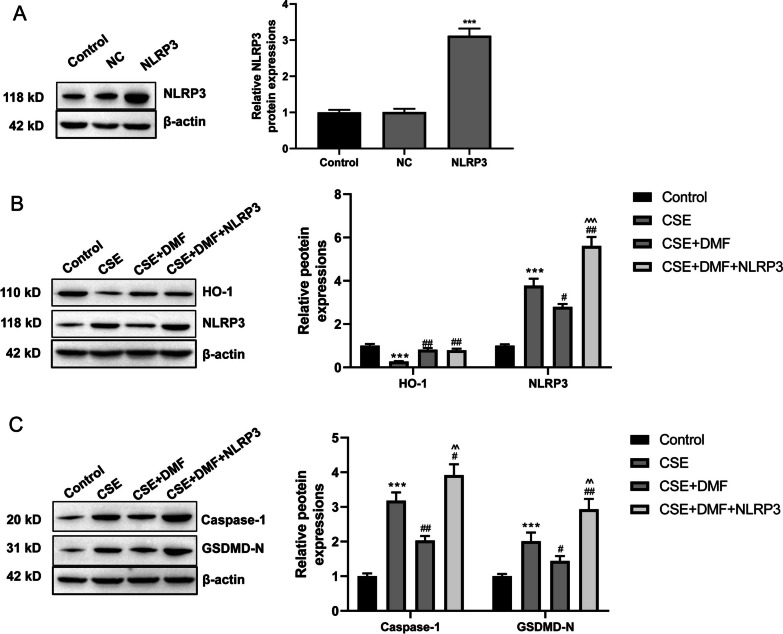
NLRP3 overexpression abolished the suppression of the activated Nrf2/HO-1 pathway on COPD cell pyroptosis. **A** Western blot revealed the successfully pCDNA3.1-NLRP3 vector-transfected NHBE cells. **B** Western blot indicated that the pCDNA3.1-NLRP3 vector-transfection reversed the suppression of the activated Nrf2/HO-1 pathway on NLRP3 expression in COPD cells. **C** Western blot implied that NLRP3 overexpression abrogated the suppression of activated Nrf2/HO-1 pathway on the expression of pyroptosis-related proteins (Caspase-1 and GSDMD-N) in COPD cells. ***P < 0.001 vs. Control group. #* P* < 0.05 and ## *P* < 0.01 vs. CSE group. ^^ *P* < 0.01 and ^^^ *P* < 0.01 vs. CSE + DMF group

To overexpress NLRP3, this study transfected pCDNA3.1-NLRP3 vectors into NHBE cells (named the NLRP3 group). NHBE cells transfected by pCDNA3.1 vectors were utilized as the NC group, and those without transfection were regarded as the Control group. As shown in Fig. 4A, pCDNA3.1-NLRP3 vectors were successfully transfected into NHBE cells, as demonstrated by the higher expression of NLRP3 protein in the NLRP3 group relative to the Control group and the NC group (*P* < 0.001). Western blot displayed the lower expressed HO-1 protein and the higher expressed NLRP3 protein in NHBE cells of the CSE group when compared to the Control group (*P* < 0.001). A distinctly higher expressed HO-1 protein was occurred in NHBE cells of the CSE + DMF group (*P* < 0.01) and the CSE + DMF + NLRP3 group relative to the CSE group (*P* < 0.01). Considering NLRP3 protein, it decreased in the CSE + DMF group (*P* < 0.05) but increased in the CSE + DMF + NLRP3 group (*P* < 0.01), when matched to NHBE cells of the CSE group. Meanwhile, in comparison to the CSE + DMF group, NHBE cells of the CSE + DMF + NLRP3 group showed the enhanced expression of NLRP3 protein (*P* < 0.001) (Fig. 4B). Additionally, higher expression of Caspase-1 and GSDMD-N proteins was examined in NHBE cells of the CSE group relative to the Control group (*P* < 0.001). Matched to NHBE cells of the CSE group, lower expressed Caspase-1 and GSDMD-N proteins in the CSE + DMF group (*P* < 0.05,* P* < 0.05) but higher expressed Caspase-1 and GSDMD-N proteins in the CSE + DMF + NLRP3 group were presented (*P* < 0.05, *P* < 0.01). In contrast to the CSE + DMF group, NHBE cells of the CSE + DMF + NLRP3 group expressed distinctly higher Caspase-1 and GSDMD-N proteins (*P* < 0.01) (Fig. 4C). All of these data illustrated that NLRP3 overexpression counteracted the suppression of the activated Nrf2/HO-1 pathway on COPD cell pyroptosis.

## Discussion

The present work demonstrated that inactivated Nrf2/HO-1 pathway and the activated NLRP3, Caspase-1 and GSDMD-N expression in COPD mouse model and cell model. Mechanistic research indicated that the activated Nrf2/HO-1 pathway might alleviate inflammation in COPD by suppressing the NLRP3-mediated pyroptosis.

Nrf2 is one of the main transcription factors, which exerts the anti-oxidative stress role by triggering the expression of the downstream anti-oxidative genes [23]. Previous studies have been identified that the activated Nrf2 possesses the function of restrict the oxidative stress and inflammatory reaction by activating its downstream HO-1 [24, 25]. As we know, HO-1 is an important antioxidant molecule possessing anti-oxidative and anti-inflammatory functions [26]. The Nrf2/HO-1 pathway is thus considered to be a crucial antioxidant defense system against multiple diseases [27]. The suppressed expression and activity of Nrf2 and HO-1 has been revealed in COPD mouse model [23] and some drugs, such as metformin and alantolactone, have been shown to relieve COPD by activating the Nrf2/HO-1 pathway [22, 28]. Similarly, this study revealed the inactivated Nrf2/HO-1 pathway in COPD mice.

More interestingly, this paper discovered the increased expression of NLRP3 in the lung tissues of COPD mice. NLRP3 is one of the key inflammasome sensors. It can trigger the activation of Caspase-1 through assembling an intact inflammasome complex, ultimately inducing the release of pro-inflammatory cytokines, such as IL-1β, tumor necrosis factor α, IL-18 and IL-6 [29, 30]. Nrf2 has ben researched to be required for the NLRP3 activation [31]. In some inflammation-related diseases, the suppressed Nrf2/HO-1 pathway has been discovered to stimulate the activation of NLRP3 to exacerbate the inflammatory response [26, 31, 32]. The activated NLRP3 has been identified in COPD, which is essential for the progression of COPD [33, 34]. However, whether the Nrf2/HO-1 pathway mediates the NLRP3 expression to regulating the development of COPD has yet to be elucidated. In this study, the activated Nrf2/HO-1 pathway was firstly demonstrated to suppress the expression of NLRP3 to relieve the inflammatory response and lung injury in COPD.

Previously study has been researched that the activated NLRP3 can trigger the pyroptosis via inducing Caspase-1 to induce the inflammatory response in some diseases [35, 36]. In the CSE-induced COPD cell model, the activated NLRP3/Caspase-1 pathway has been suggested to induce the pyroptosis to trigger the inflammatory response [37]. GSDMD-N is the ultimate executor of pyroptosis and pyroptosis often occurs upon Caspase-1 activation, because the activated Caspase-1 can cleavage GSDMD protein into GSDMD-N to form holes on the cell membrane to induce the release of proinflammatory factors [38]. The activated NLRP3/Caspase-1/GSDMD pathway has been implied to facilitate the development of several diseases by enhancing the pyroptosis [39, 40]. Similarly, this research revealed the activated NLRP3/Caspase-1/GSDMD-N pathway in the COPD mouse model and cell model. More importantly, this paper demonstrated that the activated Nrf2/HO-1 pathway could relieve the lung injury in COPD mice and suppress the expression of NLRP3, Caspase-1 and GSDMD-N and the release of proinflammatory factors (IL-1β and IL-6) in COPD mouse model and cell model. Conversely, after the Nrf2/HO-1 pathway being inactivated, an opposite results were discovered. Taken together, the activated Nrf2/HO-1 pathway might attenuate the lung injury and inflammatory response in COPD by inhibiting the pyroptosis via suppressing the NLRP3/Caspase-1/GSDMD-N.

## Conclusion

This study revealed the remitting effect of the activated Nrf2/HO-1 pathway in COPD. The activated Nrf2/HO-1 pathway might relieve the inflammatory response in COPD by suppressing the NLRP3-mediated pyroptosis. This mechanism was firstly elucidated in COPD. Activating the Nrf2/HO-1 pathway may be a promising way to treat COPD. In the future research, attention should be devoted to developing drugs that target activating the Nrf2/HO-1 pathway.

## Data Availability

The datasets used or analyzed during the current study are available from the corresponding author on reasonable request.

## References

[1] Uwagboe I (2022). New drugs under development for COPD. Minerva Med.

[2] Yang W (2021). Focus on early COPD: definition and early lung development. Int J Chron Obstruct Pulmon Dis.

[3] Christenson SA (2022). Chronic obstructive pulmonary disease. Lancet.

[4] Guo P (2022). Pathological mechanism and targeted drugs of COPD. Int J Chron Obstruct Pulmon Dis.

[5] Sikjær MG (2022). Parental COPD as a risk factor for the development of COPD and disease severity in offspring: a systematic scoping review. Int J Chron Obstruct Pulmon Dis.

[6] Chen ZY (2022). Effect of doxofylline on pulmonary inflammatory response and oxidative stress during mechanical ventilation in rats with COPD. BMC Pulm Med.

[7] Williams D (2022). The role of the pharmacist in optimizing outcomes with roflumilast, a PDE4 inhibitor for the treatment of COPD. J Pharm Pract.

[8] Feng Y (2022). Pyroptosis in inflammation-related respiratory disease. J Physiol Biochem.

[9] Wang L (2021). TREM-1 aggravates chronic obstructive pulmonary disease development via activation NLRP3 inflammasome-mediated pyroptosis. Inflamm Res.

[10] Mo R (2022). Nicotine promotes chronic obstructive pulmonary disease via inducing pyroptosis activation in bronchial epithelial cells. Mol Med Rep.

[11] Xu P (2021). TREM-1 exacerbates neuroinflammatory injury via NLRP3 inflammasome-mediated pyroptosis in experimental subarachnoid hemorrhage. Transl Stroke Res.

[12] Mahalanobish S (2020). Melatonin induced suppression of ER stress and mitochondrial dysfunction inhibited NLRP3 inflammasome activation in COPD mice. Food Chem Toxicol.

[13] Rumora L (2021). Pathogen-associated molecular patterns and extracellular Hsp70 interplay in NLRP3 inflammasome activation in monocytic and bronchial epithelial cellular models of COPD exacerbations. APMIS.

[14] Wen J (2021). Tetramethylpyrazine nitrone improves motor dysfunction and pathological manifestations by activating the PGC-1α/Nrf2/HO-1 pathway in ALS mice. Neuropharmacology.

[15] Lee J (2021). An update on the role of Nrf2 in respiratory disease: molecular mechanisms and therapeutic approaches. Int J Mol Sci.

[16] Lin Y (2020). Gallic acid alleviates gouty arthritis by inhibiting NLRP3 inflammasome activation and pyroptosis through enhancing Nrf2 signaling. Front Immunol.

[17] Lu W (2018). Hydrogen gas inhalation protects against cigarette smoke-induced COPD development in mice. J Thorac Dis.

[18] Dang R (2022). Edaravone ameliorates depressive and anxiety-like behaviors via Sirt1/Nrf2/HO-1/Gpx4 pathway. J Neuroinflamm.

[19] Qiu YB (2020). Nrf2 protects against seawater drowning-induced acute lung injury via inhibiting ferroptosis. Respir Res.

[20] Shigemura M (2018). Hypercapnia increases airway smooth muscle contractility via caspase-7-mediated miR-133a-RhoA signaling. Sci Transl Med.

[21] Han G (2021). Nucleotide-oligomerizing domain-1 activation exaggerates cigarette smoke-induced chronic obstructive pulmonary-like disease in mice. Int J Chron Obstruct Pulmon Dis.

[22] Dang X (2020). Alantolactone suppresses inflammation, apoptosis and oxidative stress in cigarette smoke-induced human bronchial epithelial cells through activation of Nrf2/HO-1 and inhibition of the NF-κB pathways. Respir Res.

[23] Cui W (2018). Nrf2 attenuates inflammatory response in COPD/emphysema: crosstalk with Wnt3a/β-catenin and AMPK pathways. J Cell Mol Med.

[24] Kundu JK, Surh YJ (2010). Nrf2-Keap1 signaling as a potential target for chemoprevention of inflammation-associated carcinogenesis. Pharm Res.

[25] Chen HH (2012). 4-Ketopinoresinol, a novel naturally occurring ARE activator, induces the Nrf2/HO-1 axis and protects against oxidative stress-induced cell injury via activation of PI3K/AKT signaling. Free Radic Biol Med.

[26] Chen Z (2019). Inhibition of Nrf2/HO-1 signaling leads to increased activation of the NLRP3 inflammasome in osteoarthritis. Arthritis Res Ther.

[27] Uddin MJ (2021). Pharmacotherapy against oxidative stress in chronic kidney disease: promising small molecule natural products targeting Nrf2-HO-1 signaling. Antioxidants (Basel).

[28] Tao F (2022). Metformin alleviates chronic obstructive pulmonary disease and cigarette smoke extract-induced glucocorticoid resistance by activating the nuclear factor E2-related factor 2/heme oxygenase-1 signaling pathway. Korean J Physiol Pharmacol.

[29] Jin C (2011). NLRP3 inflammasome plays a critical role in the pathogenesis of hydroxyapatite-associated arthropathy. Proc Natl Acad Sci U S A.

[30] Place DE, Kanneganti TD (2018). Recent advances in inflammasome biology. Curr Opin Immunol.

[31] Li D (2020). Zinc promotes functional recovery after spinal cord injury by activating Nrf2/HO-1 defense pathway and inhibiting inflammation of NLRP3 in nerve cells. Life Sci.

[32] Xue R (2021). Lycopene alleviates hepatic ischemia reperfusion injury via the Nrf2/HO-1 pathway mediated NLRP3 inflammasome inhibition in Kupffer cells. Ann Transl Med.

[33] Yang W (2015). NLRP3 inflammasome is essential for the development of chronic obstructive pulmonary disease. Int J Clin Exp Pathol.

[34] Nachmias N (2019). NLRP3 inflammasome activity is upregulated in an in-vitro model of COPD exacerbation. PLoS ONE.

[35] Sun L (2019). Propofol directly induces caspase-1-dependent macrophage pyroptosis through the NLRP3-ASC inflammasome. Cell Death Dis.

[36] Qiu Z (2019). Lipopolysaccharide (LPS) aggravates high glucose- and hypoxia/reoxygenation-induced injury through activating ROS-dependent NLRP3 inflammasome-mediated pyroptosis in H9C2 cardiomyocytes. J Diabetes Res.

[37] Zhang MY (2021). Cigarette smoke extract induces pyroptosis in human bronchial epithelial cells through the ROS/NLRP3/caspase-1 pathway. Life Sci.

[38] Kang R (2018). Lipid peroxidation drives gasdermin D-mediated pyroptosis in lethal polymicrobial sepsis. Cell Host Microbe.

[39] Li S (2021). NLRP3/caspase-1/GSDMD-mediated pyroptosis exerts a crucial role in astrocyte pathological injury in mouse model of depression. JCI Insight.

[40] Zhang Y (2022). Downregulated XBP-1 rescues cerebral ischemia/reperfusion injury-induced pyroptosis via the NLRP3/Caspase-1/GSDMD axis. Mediators Inflamm.

